# Efficacy of High-Dose Synbiotic Additives for Deoxynivalenol Detoxification: Effects on Blood Biochemistry, Histology, and Intestinal Microbiome in Weaned Piglets

**DOI:** 10.3390/biology13110889

**Published:** 2024-10-31

**Authors:** Jin-Young Jeong, Junsik Kim, Minji Kim, Sungkwon Park

**Affiliations:** 1 Animal Nutrition and Physiology Division, National Institute of Animal Science, Wanju 55365, Republic of Korea; kkk940326@korea.kr (J.K.); mjkim00@korea.kr (M.K.); 2 Department of Food Science and Biotechnology, Sejong University, Seoul 05006, Republic of Korea; sungkwonpark@sejong.ac.kr

**Keywords:** apoptosis, deoxynivalenol, synbiotic, fibrosis, piglet

## Abstract

Deoxynivalenol (DON) is a common mycotoxin observed in cereal grains, and feed contamination poses health risks to pigs. Biological antidotes, such as synbiotics (SYNs), have garnered attention for mitigating DON toxicity. This study aimed to assess the efficacy of SYNs by comparing the blood biochemistry, histology, and gut microbiome of weaned piglets. A 4-week trial was conducted on 32 weaned piglets. After a week of diet and environmental adaptation, the pigs were divided into four groups: (1) control (CON, *n* = 8); (2) SYN (*n* = 8); (3) DON (*n* = 8); and (4) DON+SYN (*n* = 8). The SYN supplementation of weaned piglets increased the final body weight (21.71 ± 0.93 vs. 20.73 ± 0.84), average daily gain (0.38 ± 0.02 vs. 0.34 ± 0.02), and gain-to-feed ratio (0.49 ± 0.04 vs. 0.43 ± 0.02), and decreased the feed conversion ratio (2.14 ± 0.14 vs. 2.39 ± 0.13) compared to the DON group. A high dose of DON induced liver and colon fibrosis and liver and cecum apoptosis, which were alleviated by SYNs. Glucose in the DON group (84.9 ± 3.7) was significantly lower than in the control (101.3 ± 4.2). Additionally, both the DON and DON+SYN groups exhibited higher creatine (0.9 ± 0.0 and 0.9 ± 0.1) and lower cholesterol (88.3 ± 3.2 and 90.0 ± 4.8) levels (*p* < 0.05). In conclusion, SYNs alleviated DON toxicity, indicating its potential as an antidote for specific biomarkers.

## Simple Summary

Mycotoxins, secondary metabolites produced by fungi, frequently contaminate feed worldwide. Specifically, deoxynivalenol (DON), a type B trichothecene produced by the Fusarium species, is the most prevalent mycotoxin detected in feed. This study using piglets evaluated the biological antidotes to mitigating DON toxicity. Our results showed that the impact of synbiotic additives on growth characteristics, histological alterations, and microbiota composition was pronounced in the DON-contaminated piglet group. Piglets fed SYN supplementation exhibited growth improvement and histological recuperation, including fibrosis and apoptosis in specific organs. Moreover, the two independent variables such as *Prevotella* 1 and *Romboutsia* were statistically significant for predicting the final BW. Furthermore, this study builds on previous research and provides insights into the influence of microbiota composition on DON detoxification. Our findings suggest the control of risks associated with DON in feed, and this study significantly contributes to the existing literature.

## 1. Introduction

Mycotoxins, secondary metabolites produced by fungi, frequently contaminate feed worldwide and adversely affect the health and survival of livestock [1]. Eighty-eight percent of the feed samples was contaminated with at least one mycotoxin [2]. Deoxynivalenol (DON), a type B trichothecene produced by *Fusarium* species, is the most prevalent mycotoxin detected in feed [3]. DON has been detected in 75.2% of feed samples from the European Union and 94% of 707 feed samples from the United States [4,5]. In Asia, DON contamination has been observed in 96.4% of 3507 feed samples from China and 95% of 494 feed samples from South Korea [6,7]. Additionally, high doses of DON can cause symptoms similar to those caused by ionizing radiation, including abdominal distress, excessive salivation, discomfort, vomiting, leukocytosis, and gastrointestinal bleeding [8].

Although all farm animals pose a risk of DON toxicity, pigs exhibit specific susceptibility owing to their heavy grain diets and lack of rumen microorganisms necessary for the breakdown of mycotoxins [8,9,10]. In general, pigs are most susceptible to DON toxicity in feed, followed by mice, rats, poultry, and ruminants [11]. Pigs are most susceptible to DON toxicity due to their high bioavailability (absolute oral bioavailability: 52.7–100%) [12], phase II glucuronidation metabolism, urinary excretion, and long elimination time [13]. Therefore, pigs exhibit an 89% DON absorption rate, making them the most sensitive farm animals [10]. High doses of DON can cause vomiting and diarrhea and reduce the growth performance of pigs, resulting in economic losses to farming [14,15,16]. Additionally, DON can induce oxidative stress by generating excessive reactive oxygen species (ROS), resulting in histological alterations, such as fibrosis and apoptosis [17,18,19]. Moreover, the exposure to DON toxicity affects various systemic metabolic processes in pigs, including glycolysis, protein biosynthesis, cell metabolism, and microbial metabolic disorders [20,21,22,23,24]. Therefore, to alleviate these effects, it is essential that we develop a mycotoxin antidote that can reduce toxicity and be used in animal feed formulations [20].

Various antidotes have been developed to mitigate DON toxicity. However, physical methods, such as adsorption, and chemical methods, such as oxidizing agents and aldehydes, may not be suitable because of the high stability of DON and the possibility of nutrient loss [25,26]. In contrast, biological antidotes have become increasingly popular as effective and environmentally friendly alternatives for mitigating the effects of DON toxicity [26,27,28]. Specifically, microbial strains are promising biological DON detoxifiers. After DON ingestion, the gut of pigs is a crucial target organ because it serves as a physical barrier to feed contaminants and enteric pathogens [8,29]. Probiotics may alleviate the adverse effects of DON ingestion by enhancing gut health, stimulating the immune system, and maintaining a balanced gut microbiome [30]. Additionally, prebiotics facilitate the growth of beneficial intestinal bacteria, and, when combined with probiotics, they can further enhance the growth and colonization of microorganisms in the intestine [31,32]. Bamboo shoots stimulate Lactobacillus acidophilus and increase short chain fatty acid (SCFA) production [33,34]. Orange peel is a promising source of cellooligosaccharides (COSs), which effectively stimulate Lactobacillus acidophilus and promote cell growth [35,36]. This combination of prebiotics and probiotics is called synbiotics (SYNs) [37,38]. However, to the best of our knowledge, there is limited research on the use of SYNs as microbial additives for DON detoxification in pigs. This study used SYNs as an antidote to minimize the effects of DON.

The toxic effects of DON may be more severe in weaned piglets than in growing-finishing pigs because of weaning stress [39,40]. Post-weaning DON exposure can significantly affect the growth and survival of weaned piglets by worsening the gut microbiota imbalances caused by rapid dietary alterations [41,42]. Therefore, we hypothesized that SYNs may be more effective in alleviating high-dose DON toxicity in weaned piglets. To assess the efficacy of SYNs, we compared the growth performance, blood biochemistry, histological alterations, and intestinal microbiome of high-dose DON-fed weaned piglets with those of microbial-additive-fed pigs.

## 2. Materials and Methods

### 2.1. Ethics Statements

All the experimental procedures were reviewed and approved by the Institutional Animal Care and Use Committee of the National Institute of Animal Science, Republic of Korea (NIAS2020-0472).

### 2.2. Animal and Study Design

Male castrated pigs were obtained from Taeheung (Yeonggwang, Republic of Korea). Thirty-two piglets (Landrace × Yorkshire × Duroc, 11.1 ± 0.2 kg) were housed in individual pens (1.3 × 2.45 m). During the study, including acclimatization, the pigs were housed under a 12:12 h light–dark cycle at a room temperature of 27 ± 1 °C and relative humidity of 70 ± 5%, according to the growth period. The pigs were divided into four distinct groups—(1) CON: control, basal diet; (2) SYN: basal diet + synbiotics (0.2% bamboo + 0.8% orange peel/kg feed, 10^10^ colony-forming units [CFU] of *Lactobacillus acidophilus* + 10^9^ *Devosia insulae*/animal); (3) DON: basal diet + deoxynivalenol (10 mg/kg feed); and (4) DON+SYN: basal diet + deoxynivalenol (10 mg/kg feed) + synbiotics (0.2% bamboo + 0.8% orange peel/kg feed, 10^10^ CFU of *Lactobacillus acidophilus* + 10^9^ *Devosia insulae*/animal). The basal feed was purchased from Cheonhajeil (Iksan, Korea). The composition of the feed ingredients is as follows: crude protein (19%), crude fat (6.0%), crude fiber (3.0%), crude ash (7.0%), calcium (0.6%), phosphorus (1.0%), and L-lysine (1.25%). Feed nutrition included digestible crude protein (16%) and digestible energy (3.6 Mcal/kg). The animals had access to food and water ad libitum throughout the study period. DON (TripleBond, Guelph Ontario, Canada) was mixed into the diet following the established experimental concentrations. The mycotoxin was dissolved in ethyl alcohol (used in alcoholic beverages) at 1–5% of the diet volume in a fully sterilized beaker and stirred until it dissolved completely. Preliminary testing determined the appropriate amount of solvent to ensure that it did not interfere with the diet fluidity based on its moisture content. The dissolved toxins were mixed in the diet using a blender to achieve a uniform mixture. DON content in the feed was analyzed using ultra-performance liquid chromatography (UPLC). A homogenized DON sample (1 g) was extracted with 20 mL distilled water, shaken for 30 min, filtered through Whatman No. 1 paper, and diluted with phosphate-buffered saline (PBS) solution. UPLC and mass spectrometry were performed as described previously [43]. DON concentration in the mixed feed was 9.61 mg/kg. The basal diet was below the limits of detection and quantification. The pigs were fed DON-contaminated feed for 28 days. Blood was collected one day before tissue sampling at the end of the experimental period. All animals were anesthetized using T61. Following exsanguination, the cecum, colon, ileum, liver, rectum, and feces were rapidly collected. The samples were rapidly frozen in liquid nitrogen and stored at −80 °C. Additionally, for histological analysis, tissues were fixed in 10% neutral buffered formalin (NBF; Sigma-Aldrich, St. Louis, MO, USA). To determine the average daily gain (ADG), average daily feed intake (ADFI), and feed conversion ratio (FCR), the following formulas were used: ADG = (final weight − initial weight)/age (days), ADFI = amount of feed provided − amount of feed remaining, and FCR = feed intake/average daily gain.

### 2.3. Blood Biochemical Analysis

Blood samples were collected from the jugular vein of each pig in anticoagulant-free vacuum tubes. The experimental method and 15 parameter analysis were performed as described previously [44]. The blood samples were centrifuged (700× *g*, 15 min, 4 °C) to collect serum and that was subsequently stored at −80 °C. Each parameter was analyzed using a VetTest chemistry analyzer (IDEXX, Westbrook, ME, USA), following the manufacturer’s protocol.

### 2.4. Histological Analysis

Fibrosis and apoptosis were observed in the cecum, colon, ileum, liver, and rectal tissues, confirming the effects of DON-detoxifying agents. Histological analysis was performed as described previously [44]. The analysis procedure involved fixing, dehydrating, and embedding the tissue samples. Subsequently, sections were cut and placed on a slide warmer. For staining, the sections were deparaffinized, rehydrated, rinsed, and stained with Masson’s trichrome (MT) to visualize fibrous connective tissue. Apoptotic cells in the liver and cecum were detected through terminal deoxynucleotidyl transferase dUTP nick-end labeling (TUNEL) using an in situ Cell Death Detection Kit (POD), following the manufacturer’s protocol. The stained sections were examined under a microscope (Micrometrics^TM^ ; Nikon ECLIPSE E200, Tokyo, Japan) at 200× *g* magnification.

### 2.5. DNA Extraction and Intestinal Microbiome

Metagenomic DNA was extracted from broiler cecal samples using the bead-beating (repeated bead-beating plus column) method [45] with a QIAamp DNA kit (Qiagen, Hilden, Germany). Artificial sequences and low-quality bases in the generated reads were removed using Trimmomatic and TruSeq3-PE with the following parameters: fa:2:30:10:2:True, LEADING:5, TRAILING:20, and MINLEN:250 [46]. After quality control of the raw data, the filtered reads were analyzed using QIIME2 [47]. The remaining adapter sequences in the filtered reads were removed using the cutadapt module in QIIME2 with -p-front-f CCTACGGGNGGCWGCAG and -p-front-r GACTACHVGGGTATCTAATCC parameters [48]. Denoising was conducted using dada2, a denoise-paired module in QIIME2, with parameters –p-trunc-len-f 230 and –p-trunc-len-r 220 [49]. Taxonomic assignment was conducted using the classify-sklearn module with a pre-trained silva-138-99-nb-classifer.qza provided by QIIME2 [50]. Following the taxonomic assignment, mitochondrial and chloroplast taxa and those whose assigned levels did not represent the minimum phylum were filtered out.

### 2.6. Statistical Analyses

The align-to-tree-mafft-fasttree module [51] was used to construct a tree for the representative amplicon sequence variant (ASV), and alpha and beta diversities were calculated using the diversity module in QIIME2 [52]. For functional pathway prediction of the microbial community, PICRUST2 was used with a frequency table exported from QIIME2 [53]. Principal component analysis (PCA) plots and statistical tests for the predicted pathways were conducted using STAMP with the Kruskal–Wallis test [54]. Differential abundance taxon analyses were performed using linear discriminant analysis effect size (LEfSe) [55]. Stepwise regression using the REG procedure and STEPWISE option of SAS was used to develop equations predicting body weight (BW) based on microbiota abundance in various intestinal contents. Significant differences in blood results and growth performance were determined at *p* < 0.05, using Prism ver. 9 software.

## 3. Results

### 3.1. Growth Performances

The effects of DON intake and SYN supplementation on the BW of weaned piglets over 28 days are summarized in Table 1. The initial BWs were similar across all dietary treatment groups. The DON (20.73 ± 0.84 kg) group had the lowest final BW compared to all treatment groups (*p* < 0.05). On day 28, the DON+SYN (21.71 ± 0.93 kg) group had a significantly lower final BW compared to the CON (22.89 ± 1.17 kg) and SYN (25.69 ± 1.17 kg) groups (*p* < 0.05). ADG was gradually reduced: SYN (0.52 ± 0.03) > CON (0.42 ± 0.04 kg) > DON+SYN (0.38 ± 0.02) > DON (0.34 ± 0.02). Additionally, the SYN group had the highest gain-to-feed ratio (G:F ratio, 0.66 ± 0.03) and lowest FCR (1.54 ± 0.09) compared to that of the other groups (*p* < 0.05). ADFI did not differ significantly among the dietary treatment groups.

### 3.2. Serum Biochemistry of DON- and SYN-Fed Piglets

The results of the blood sample analysis of 17 biochemical parameters are summarized in Table 2. Blood urea nitrogen (BUN), BUN/creatine (CREA), phosphate (PHOS), calcium (CA), total protein (TP), albumin globulin (ALB), globulin (GLOB), ALB/GLOB, alanine aminotransferase (ALT), alkaline phosphatase (ALKP), gamma-glutamyl transpeptidase (GGT), total bilirubin (TBIL), cholesterol (CHOL), amylase (AMYL), and lipase (LIPA) levels were not significantly different between treatments. However, glucose (GLU) levels were lowest in the DON group compared to those in the control, SYN, and DON+SYN groups (*p* < 0.05, Table 2). Additionally, the DON and DON+SYN groups had higher CREA and lower CHOL levels than the control and SYN groups (*p* < 0.05). Overall, we observed that the addition of SYNs enhanced the GLU and CHOL levels.

### 3.3. Histological Alterations in DON- and SYN-Fed Piglets

**Figure 1:**
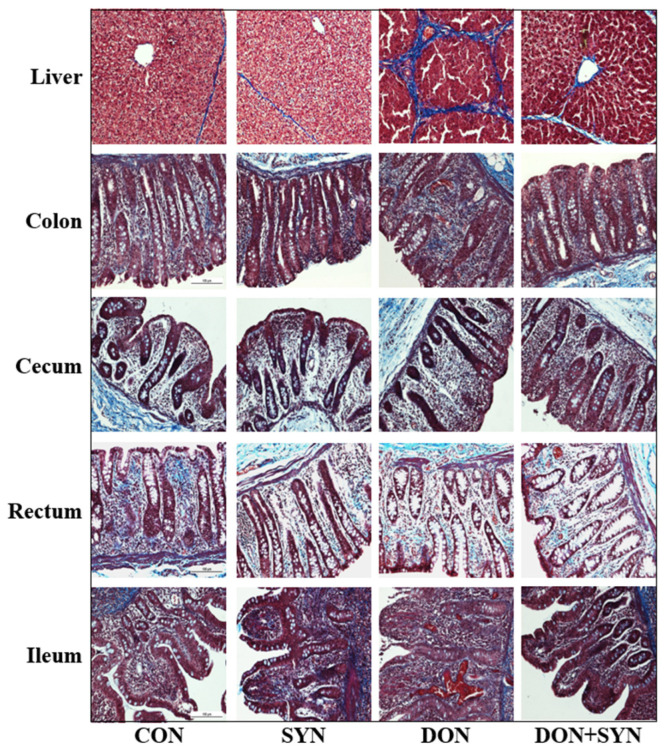
Effects of high-dose deoxynivalenol (DON) and synbiotics (SYNs) as DON-detoxifying additives on histological analysis of weaned piglets. Images of the liver, colon, cecum, rectum, and ileum of weaned piglets 28 days after the trial using Masson’s trichrome (blue) staining following DON and SYN intake. In the liver and colon tissues, high-dose DON intake resulted in increased collagen deposition, whereas SYN attenuated DON-induced collagen deposition. Treatments: CON: control, basal diet; SYN: basal diet + synbiotics (0.2% bamboo + 0.8% orange peel/kg feed, 10^10^ colony-forming units [CFU] of *Lactobacillus acidophilus* + 10^9^ *Devosia insulae*/animal); DON: basal diet + deoxynivalenol (10 mg/kg feed); and DON+SYN: basal diet + deoxynivalenol (10 mg/kg feed) + synbiotics (0.2% bamboo + 0.8% orange peel/kg feed, 10^10^ CFU of *Lactobacillus acidophilus* + 10^9^ *Devosia insulae*/animal).

**Figure 2:**
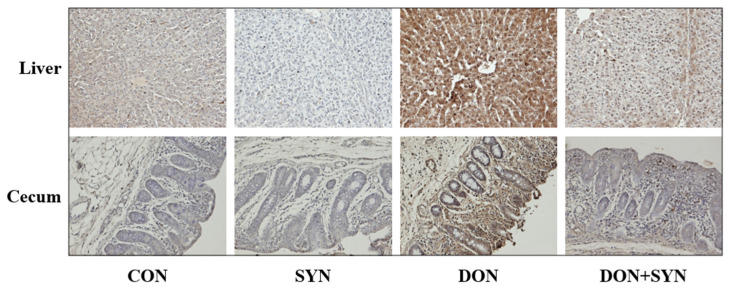
Effects of high-dose deoxynivalenol (DON) and synbiotics (SYNs) as DON-detoxifying additives on apoptosis in weaned piglets. Images of the liver and cecum of weaned piglets 28 days after trial using terminal deoxynucleotidyl transferase dUTP nick-end labeling (TUNEL) staining following DON and SYN intake. In both organs, TUNEL-positive staining increased in the DON group and decreased in the DON+SYN group. Treatments: CON: control, basal diet; SYN: basal diet + synbiotics (0.2% bamboo + 0.8% orange peel/kg feed, 10^10^ colony-forming units [CFU] of *Lactobacillus acidophilus* + 10^9^ *Devosia insulae*/animal); DON: basal diet + deoxynivalenol (10 mg/kg feed); and DON+SYN: basal diet + deoxynivalenol (10 mg/kg feed) + synbiotics (0.2% bamboo + 0.8% orange peel/kg feed, 10^10^ CFU of *Lactobacillus acidophilus* + 10^9^ *Devosia insulae*/animal).

The histological alterations in DON- and SYN-fed piglets, as indicated by MT staining and the TUNEL assay, are illustrated in Figure 1 and Figure 2. Our analysis demonstrated that the rectal and ileal tissues did not exhibit any significant histological alterations (Figure 1). However, liver cells from the DON group exhibited significant histological differences compared to those from the control and SYN groups. Piglets in the DON group demonstrated the disruption of liver structure with extensive blue staining, indicating severe fibrosis and a high collagen deposition between the connective tissues of the hepatic lobules (Figure 1). This group exhibited significant fibrosis with dense fibrotic bands and the nodular regeneration of the liver tissue, with the portal vein, artery, and bile duct stained blue by MT. Additionally, blue staining was observed around the lobular border, where enlarged portal veins and increased DON accumulation were observed. The DON+SYN group exhibited a significant enhancement compared to that of the DON group, with reduced collagen deposition, although some blue staining remained. Additionally, histological alterations were observed in the colonic tissues of piglets (Figure 1). The DON group exhibited increased collagen deposition throughout the colonic mucosa and submucosal glands compared to that of the control and SYN groups (Figure 1). However, the SYN group exhibited significantly less staining for fibrosis compared to that of the DON group (Figure 1). Moreover, the DON group exhibited a significant increase in TUNEL-positive staining throughout the liver and cecum tissues compared to that of the control and SYN groups, indicating severe apoptosis (Figure 2). In contrast, the DON+SYN group exhibited higher TUNEL-positive staining than that of the control and SYN groups, but lower than that of the DON group (Figure 2).

### 3.4. Intestinal Microbiome in DON- and SYN-Fed Piglets

**Figure 3:**
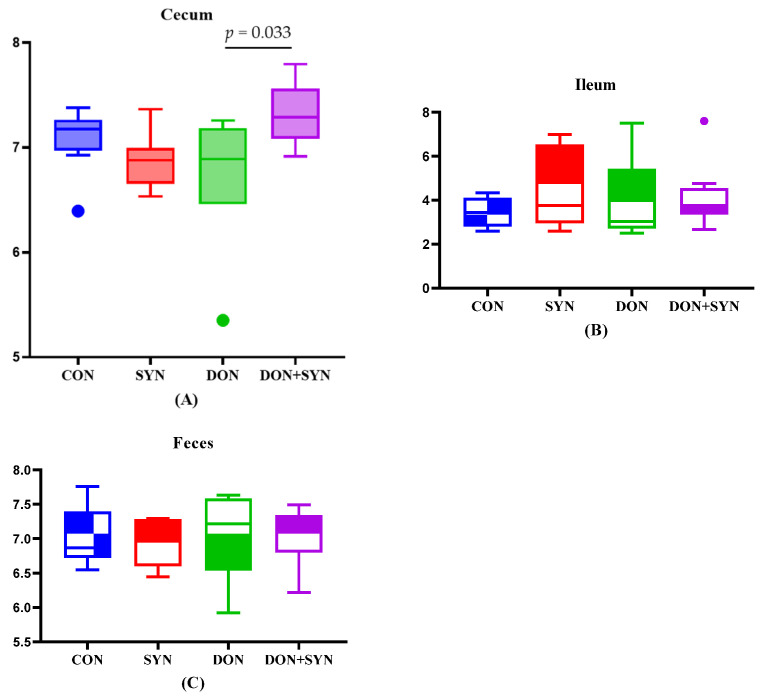
Alpha diversity analysis using the Shannon index to analyze the diversity of the intestinal microbiome based on deoxynivalenol (DON) and synbiotic (SYN) intake. (**A**) Cecum, (**B**) ileum, and (**C**) feces. The results exhibited no significant differences in alpha diversity of ileum and fecal tissues among the four dietary treatments. Treatments: CON: control, basal diet; CON+SYN: basal diet + synbiotics (0.2% bamboo + 0.8% orange peel/kg feed, 10^10^ colony-forming units [CFU] of *Lactobacillus acidophilus* + 10^9^ *Devosia insulae*/animal); DON: basal diet + deoxynivalenol (10 mg/kg feed); and DON+SYN: basal diet + deoxynivalenol (10 mg/kg feed) + synbiotics (0.2% bamboo + 0.8% orange peel/kg feed, 10^10^ CFU of *Lactobacillus acidophilus* + 10^9^ *Devosia insulae*/animal). Outliers are shown as dots.

The alpha diversity, assessed using the Shannon index, was measured to analyze the diversity of intestinal microbiota in response to DON and SYN ingestion (Figure 3). The results exhibited no significant differences in diversity among the dietary groups, except for the cecum (*p* < 0.05, Figure 3).

**Figure 4:**
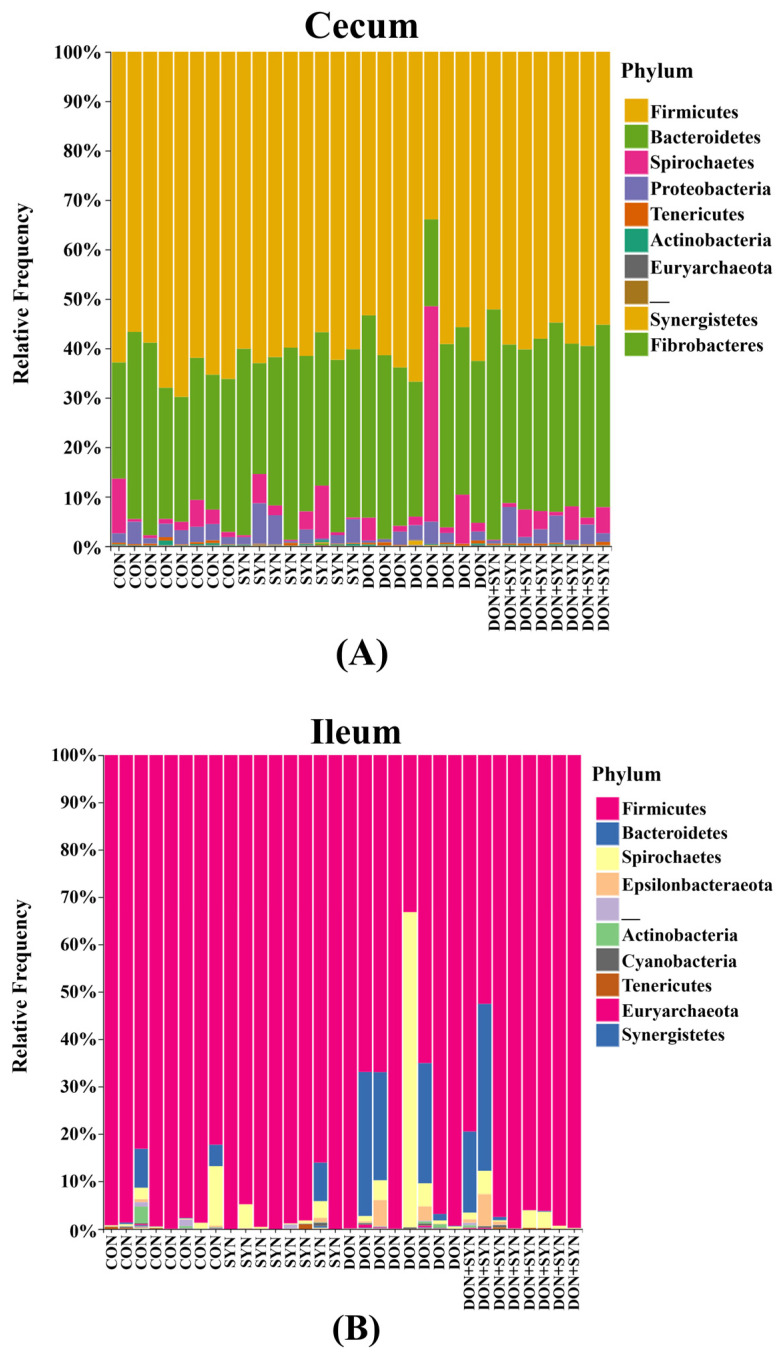

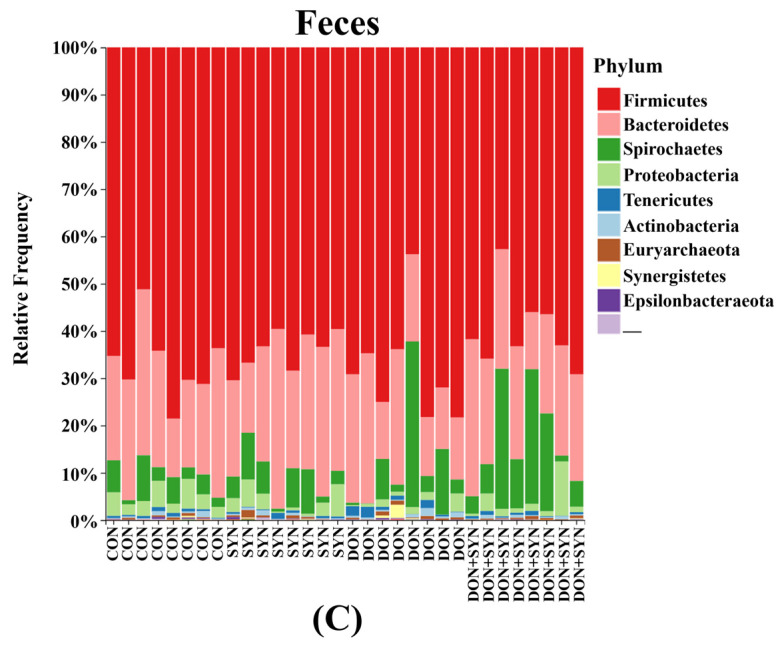
Microbial taxonomic bar plot from the (**A**) cecum, (**B**) ileum, and (**C**) feces of weaned piglets from the four dietary treatments at the phylum level. Taxonomic compositions of the microbiota among the four dietary treatments are compared based on the relative abundance (taxon reads/total reads in the cecum, ileum, and feces). Treatments: CON: control, basal diet; DON+SYN: basal diet + synbiotics (0.2% bamboo + 0.8% orange peel/kg feed, 10^10^ colony-forming units [CFU] of *Lactobacillus acidophilus* + 10^9^ *Devosia insulae*/animal); DON: basal diet + deoxynivalenol (10 mg/kg feed); and DON+SYN: basal diet + deoxynivalenol (10 mg/kg feed) + synbiotics (0.2% bamboo + 0.8% orange peel/kg feed, 10^10^ CFU of *Lactobacillus acidophilus* + 10^9^ *Devosia insulae*/animal).

**Figure 5:**
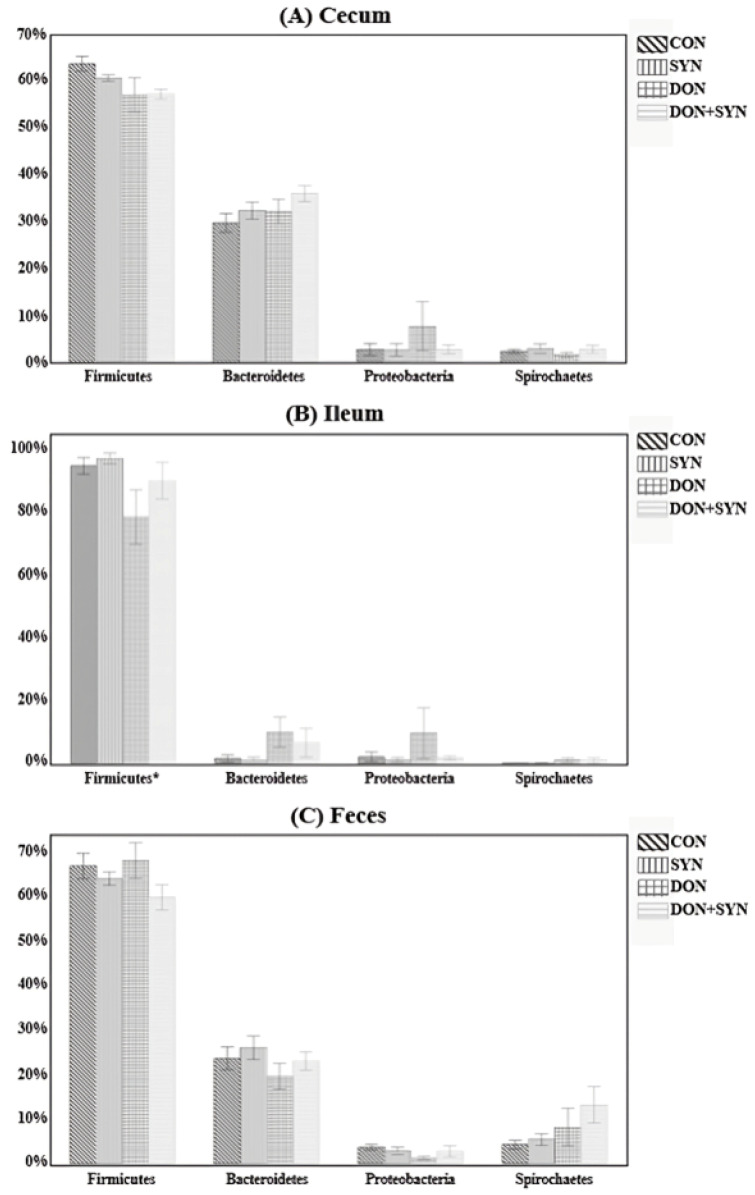
Relative abundance of the microbiome in the cecum (**A**), feces (**B**), and ileum (**C**) at the phylum level among the four dietary treatment groups. Treatments: CON: control, basal diet; CON+SYN: basal diet + synbiotics (0.2% bamboo + 0.8% orange peel/kg feed, 10^10^ colony-forming units [CFU] of *Lactobacillus acidophilus* + 10^9^ *Devosia insulae*/animal); DON: basal diet + deoxynivalenol (10 mg/kg feed); and DON+SYN: basal diet + deoxynivalenol (10 mg/kg feed) + synbiotics (0.2% bamboo + 0.8% orange peel/kg feed, 10^10^ CFU of *Lactobacillus acidophilus* + 10^9^ *Devosia insulae*/animal). * *p* < 0.1.

The microbial composition in the cecum, ileum, and feces was determined using the 16S rRNA sequencing of weaned piglets. A total of 1,354,437 sequences were generated from the cecum, 1,573,993 from the ileum, and 2,060,748 from the feces. Additionally, taxonomic bar plots demonstrating the mean relative abundance at the phylum level for all four dietary treatments are illustrated in Figure 4. Firmicutes was the most abundant phylum in the cecum (59.8%), ileum (64.7%), and feces (89.8%) (Figure 4), followed by Bacteroidetes in the cecum (32.5%), ileum (23.1%), and feces (5.3%) (Figure 4). Spirochaetes ranked third in the cecum (4.4%) and ileum (7.8%), whereas Proteobacteria ranked third in the feces (3.6%). In the ileum, the Firmicutes phylum was lower in the DON group than in the other groups (94.88 vs. 97.18 vs. 78.51 vs. 90.10%, respectively; *p* = 0.0972; Figure 5), whereas the other phyla exhibited no significant differences among the dietary treatment groups. Conversely, in the cecum and feces, no significant differences were observed among the dietary treatment groups for all phyla.

**Figure 6:**
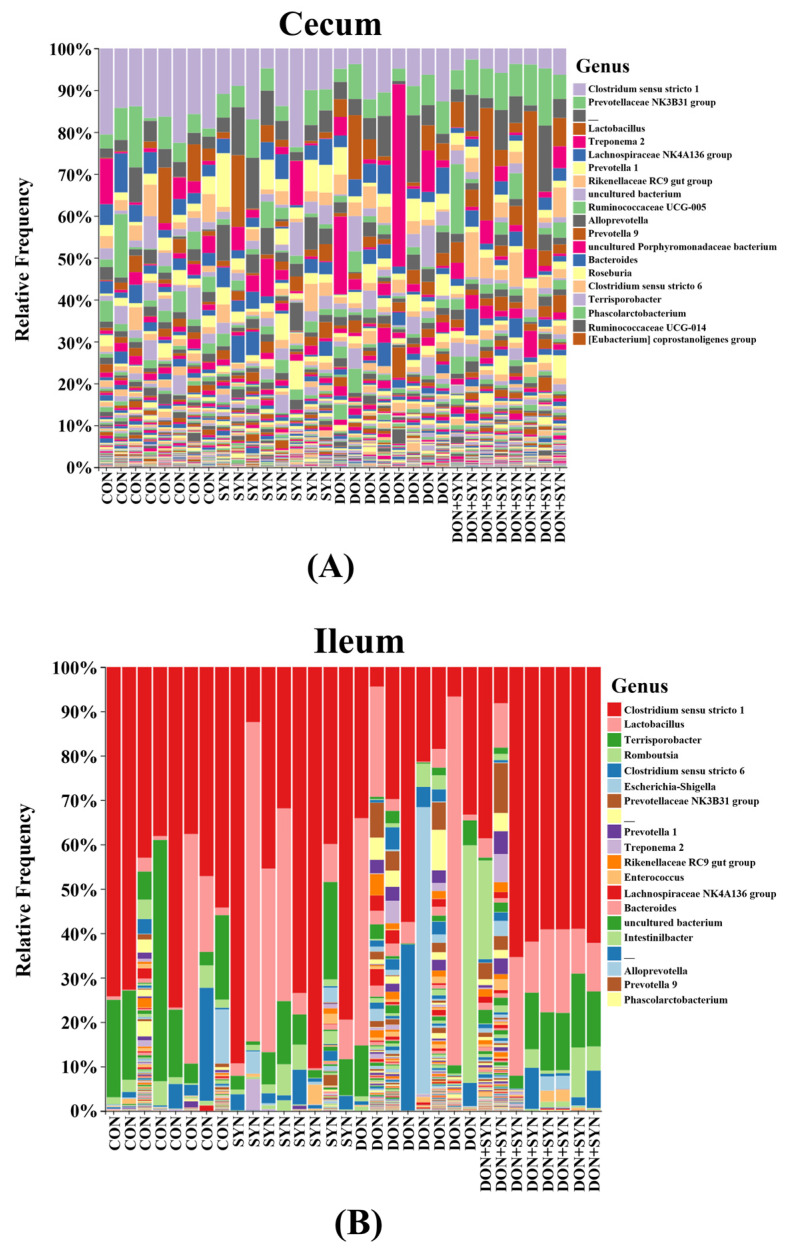

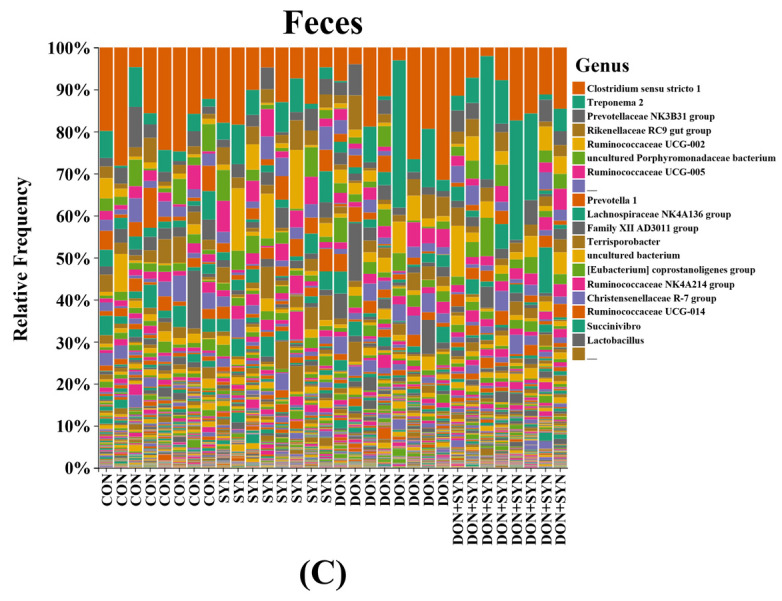
Microbial taxonomic bar plot from the (**A**) cecum, (**B**) ileum, and (**C**) feces of weaned piglets from the four dietary treatments at the genus level. Taxonomic compositions of the microbiota among the four dietary treatments are compared based on the relative abundance (taxon reads/total reads in the cecum, ileum, and feces). Treatments: CON: control, basal diet; SYN: basal diet + synbiotics (0.2% bamboo + 0.8% orange peel/kg feed, 10^10^ colony-forming units [CFU] of *Lactobacillus acidophilus* + 10^9^ *Devosia insulae*/animal); DON: basal diet + deoxynivalenol (10 mg/kg feed); and DON+SYN: basal diet + deoxynivalenol (10 mg/kg feed) + synbiotics (0.2% bamboo + 0.8% orange peel/kg feed, 10^10^ CFU of *Lactobacillus acidophilus* + 10^9^ *Devosia insulae*/animal).

Taxonomic bar plots illustrating the mean relative abundances at the genus level for all four dietary treatments are presented in Figure 6. *Clostridium sensu stricto* 1 was the most abundant genus in the cecum (10.6%), ileum (47.7%), and feces (13.9%) (Figure 6). In the cecum, other genera accounting for > 3% of the total 1,354,437 sequences included *Prevotellaceae* NK3B31 (5.8%), *Lactobacillus* (5.3%), *Treponema* 2 (4.1%), *Lachnospiraceae* NK4A136 (3.4%), and *Prevotella* 1 (3.1%) (Figure 6A). In the ileum, *Lactobacillus* (16.9%), *Terrisporobacter* (9.5%), *Romboutsia* (4.7%), and *Clostridium sensu stricto* 6 (4.3%) accounted for > 3% of the total 1,573,993 sequences (Figure 6B). In the feces, *Treponema* 2 (7.5%), *Ruminococcaceae* UCG-002 (3.2%), *Prevotellaceae* NK3B31 (3.2%), and *Rikenellaceae* RC9 gut group (3.1%) accounted for > 3% of the total 2,060,748 sequences (Figure 6C).

Stepwise multiple regression analyses were performed to identify which of the 36 genera significantly predicted the final BW (Table 3). The two independent variables were statistically significant (*p* < 0.05). The combined predictive value of the microbiota abundance of *Prevotella* 1 and *Romboutsia* was 41%. Of these, the *Romboutsia* genera exhibited the greatest contribution to the final BW (27%), followed by *Prevotella* 1 (14% of the final BW of the total variability). Therefore, *Romboutsia* appears to have the greatest effect on the final BW.

## 4. Discussion

In this study, we administered a SYN additive to weaned piglets by combining *Lactobacillus acidophilus* and *Devosia insulae* as probiotics and bamboo and orange peel as prebiotics. *Lactobacillus acidophilus* has a high toxin-removing capacity, demonstrating its potential to degrade mycotoxins or reduce their bioavailability [56,57,58]. Additionally, Gram-positive bacteria, such as *Lactobacillus,* have been observed to adsorb DON and may participate in mycotoxin–bacterial interactions, thereby facilitating toxicity reduction [17]. *Devosia insulae* is effective in biodegrading DON and its derivatives, including 3-Ac-DON and 15-Ac-DON, into the less toxic metabolite 3-keto-DON [22,23]. Bamboo, used in this study, is known for its high-quality dietary fiber and has a prebiotic effect that regulates the composition of the intestinal microbiome, thereby facilitating *Lactobacillus* growth and increased short-chain fatty acids (SCFAs) [59,60]. Additionally, orange peel is a major source of pectin oligosaccharides, supports the activity and growth of several probiotic microbiota, and increases SCFA formation [36,61]. Therefore, we anticipated that the combination of *Lactobacillus acidophilus* and *Devosia insulae* as probiotics may alleviate the DON metabolism-associated toxicity in weaned piglets. Additionally, we hypothesized that bamboo and orange peel may maximize the synergistic effects as potential prebiotics.

In this study, the intake of synthetic biological supplements enhanced growth performance, with 23.8 and 12.2% increases in ADG and final BW, respectively, compared to that of the control group. Although the ADFI did not differ significantly among the dietary treatment groups, the G:F ratio and FCR were significantly enhanced by SYN supplementation. These findings indicate that bioengineered feed enhanced feed efficiency by enhancing digestibility and nutrient absorption [62]. *Lactobacillus acidophilus*, as a probiotic, enhanced the growth performance in weaned pigs by enhancing the intestinal barrier morphology and regulating the balance of intestinal microflora, thereby enhancing nutrient absorption [15,16,63]. Additionally, supplementation with *Devosia* sp. effectively mitigated the adverse effects of DON on the growth performance of pigs [64]. Moreover, orange peel and bamboo enhanced the activity and growth of these probiotics [36,60]. Therefore, our findings highlight the potential benefits of these four biological additives as SYN for enhancing the growth performance in weaned piglets.

DON adversely affects the growth performance of weaned piglets, with growth retardation being a common symptom of DON toxicosis [14,15,16,65]. In this study, high DON concentrations reduced the daily weight gain of weaned piglets by 19% compared to that of the control group, resulting in a 9.4% reduction in final BW. Additionally, the G:F ratio increased by 17.3% and FCR decreased by 18.9%. However, SYN supplementation did not significantly alleviate these adverse effects of DON. Similar to our findings, Li et al. [66] did not observe a detoxifying effect of DON on growth performance following probiotic supplementation. In contrast, numerous studies have reported adverse effects of DON and the efficacy of biological additives in enhancing the growth performance of weaned piglets [25,27,28,67]. Our results indicate the potential benefits of SYNs on growth performance but do not demonstrate a clear effect on DON detoxification. The ability of feed additives to mitigate DON effects may vary based on factors, such as the contamination level, pig health status, and feeding duration [64,68]. The provision of synbiotics during the growth-fattening phase has a positive effect on the growth performance, carcass characteristics, and fecal microbiota in pigs [69]. Positive effects on the growth performance and blood biochemistry in pigs when fed synbiotics during the growing-finishing period [70]. The provision of a synbiotic suggests a viable approach to improving the performance, health, and welfare of pigs raised in high-temperature environments [71]. Therefore, further studies are required to consider various external and internal factors associated with DON.

In this study, the GLU, CREA, and CHOL levels were significantly affected by high doses of DON. We observed increased blood CREA levels in weaned piglets in the DON-treatment groups, which aligned with the study by Wu et al. [72], in which 6 mg/kg of DON was administered to 60-day-old pigs (BW of 16.28 kg). CREA, a byproduct of creatinine metabolism, is a significant parameter for assessing kidney damage because it reflects the degree of glomerular filtration [73,74]. High doses of DON can induce oxidative stress and disorders in kidney cells [73], which may be related to our results. Additionally, our findings demonstrated a reduction in CHOL in response to high doses of DON, indicating that DON inhibits CHOL production in weaned piglets. CHOL is a major component of lipid metabolism, and the liver is the primary organ for de novo CHOL synthesis [75]. Maintaining CHOL homeostasis is crucial for mitigating DON-induced liver damage [76]. However, mycotoxins can inhibit CHOL production by regulating the genes involved in its metabolism [75]. Moreover, our results indicated that high doses of DON inhibited GLU production in weaned piglets, whereas SYN supplementation mitigated this adverse effect, similar to the findings of Holanda and Kim [77]. The sodium–glucose dependent transporter (SGLT-1) is the major transporter responsible for GLU absorption in the small intestine and is highly sensitive to DON. Therefore, DON toxicity can impair intestinal GLU absorption [8]. SYN supplementation can address GLU control issues caused by intestinal dysfunction by enhancing the intestinal microbial environment and barrier function [78]. Liver function can be assessed by blood biochemistry, but the effect of DON toxicity on this has been inconsistent across studies [79]. Thus, liver damage caused by DON toxicity may not result in the abnormal excretion of ALT and ALKP. However, as ALT and ALKP are used as indicators of liver damage, further research is needed to elucidate the mechanism.

The liver is the primary organ affected by DON because it is responsible for detoxifying and metabolizing DON after the ingestion of contaminated feed [80]. Additionally, the intestinal tract serves as a significant target for DON-contaminated feed, acting as an initial barrier to contaminants, chemicals, and pathogens [8,29]. In this study, high doses of DON induced histological alterations in liver and intestinal tissues, including fibrosis and apoptosis, whereas SYN supplementation alleviated these adverse effects. These histological alterations may have occurred because of DON-induced oxidative stress [81]. DON causes excessive ROS production and significantly reduces antioxidant enzyme concentration and function, thereby causing cellular oxidative stress [17]. This can damage the function and structure of mitochondrial membranes, resulting in liver apoptosis [17]. Additionally, DON-induced oxidative stress increases the expression of genes associated with inflammation and apoptosis in the intestinal epithelial cells of pigs [80]. Moreover, DON-induced oxidative stress can cause fibrosis [82], characterized by the excessive accumulation of matrix connective tissue components that can affect several organs [83]. Oxidative stress can facilitate the expression of target fibrotic genes, such as tumor growth factor beta 1 (TGF-β1) that contributes to fibrosis through extracellular matrix accumulation [84,85]. Furthermore, oxidative stress can damage cells, triggering an inflammatory response, and its sustained activation can cause tissue fibrosis [84,85,86]. *Lactobacillus acidophilus* and *Devosia insulae* in the probiotics provided in this study can be considered promising antioxidant candidates because they can suppress DON-induced oxidative stress [17,87,88]. The ability to produce pyrroloquinoline quinone (PQQ) was found to be essential for the degradation of deoxynivalenol (DON) by a new bacterium, *Devosia* sp. D-G15 [89]. Additionally, bamboo and orange peel, which serve as prebiotics, have been reported to reduce oxidative stress in pigs [90,91]. Therefore, our findings indicated that the antioxidant effects of SYNs attenuated DON-induced histological alterations in weaned piglets.

The intestinal microbiota is crucial for intestinal function, and its imbalance can cause health problems in the host [10,25]. The intestinal tract is a major target organ of DON-contaminated feed [8,29]. Specifically, the adverse effects of DON on intestinal microbiota may be maximized by rapid dietary alterations in weaned piglets [22,23,92]. However, in this study, the four dietary treatments did not significantly affect the microbiota diversity of the weaned piglets, except for the cecum. These results are similar to those of Liu et al. [41,42], which reported no difference in the Shannon index for the duodenum, jejunum, and ileum of DON-fed weaned piglets. In contrast, a high dose of DON (8 mg/kg) affected the Shannon index of the cecum microbiota in pigs [43]. These contrasting results may be because of the differences in host age, DON status, DON content, feeding period, and dietary composition [93]. In this study, Firmicutes and Bacteroidetes were the most abundant phyla in the cecum, ileum, and feces across all four dietary groups. This taxonomic composition aligns with the findings of previous studies on the intestinal microbiome of pigs ingesting DON [43,93]. Additionally, our results indicated that DON reduced Firmicutes in the ileum of weaned piglets, whereas SYN supplementation alleviated this adverse effect. Similarly, DON reduced the abundance of Firmicutes in the ileum of pigs [66] and increased the abundance of Firmicutes through *Lactobacillus acidophilus* supplementation as a probiotic [94]. Firmicutes are known to exhibit a positive effect on growth by decomposing indigestible polysaccharides, thereby facilitating the digestion and absorption of nutrients in the body [95]. Our findings indicate that SYN supplementation may enhance gut health by maintaining a balance of beneficial microbiota. However, in the cecum and feces, no significant differences were observed among the four dietary treatment groups. Additionally, *Clostridium sensu stricto* 1 was the most predominant genus in the cecum, ileum, and feces. It is commonly observed in the intestines of pigs, and its abundance increases after weaning [96,97]. However, except for *Clostridium sensu stricto* 1, the dominance order among the remaining genera differed in the cecum, ileum, and feces. The gastrointestinal tract varies significantly in its nutritional and chemical compositions, water and oxygen contents, temperatures, and pH levels based on its specific location [98]. Therefore, our findings may be attributed to these regional differences in the gut microbial composition, which are affected by functional diversity. However, in this study, we highlighted the diversity in feces and various segments of the gastrointestinal tract [98].

## 5. Conclusions

Our study assessed the effects of high-dose DON on the growth performance, blood biochemistry, histology, metabolite profiling, and intestinal microbiome in weaned piglets over four weeks, and the efficacy of SYNs in alleviating DON toxicity. We observed that high doses of DON reduced the growth performance in weaned piglets. Additionally, our findings indicate that high doses of DON cause alterations in tissue-specific metabolites and histological alterations, including fibrosis and apoptosis, in specific organs, while negatively disrupting the gut microbiota balance. These toxic effects of DON were mitigated by the administration of SYNs, highlighting its promising potential as an antidote targeting specific biomarkers. However, despite these positive effects, no significant enhancement was observed in the growth performance of weaned piglets supplemented with SYNs, which may be attributed to various complex factors. Therefore, further studies considering factors, such as contamination levels, feeding duration, and the health status of pigs are necessary to fully elucidate the efficacy of SYNs in combating high-dose DON toxicity.

## Figures and Tables

**Table 1:** Effects of high-dose deoxynivalenol (DON) and synbiotics (SYNs) as DON-detoxifying additives on growth performance of weaned piglets ^1^.

	CON	SYN	DON	DON+SYN	*p* Value
Parameters ^2^	(*n* = 8)	(*n* = 8)	(*n* = 8)	(*n* = 8)	
Initial BW ^3^, kg	11.03 ± 0.39	11.03 ± 0.42	11.15 ± 0.48	11.16 ± 0.49	0.9945
Final BW ^4^, kg	22.89 ± 1.17 ^ab^	25.69 ± 1.17 ^a^	20.73 ± 0.84 ^b^	21.71 ± 0.93 ^b^	0.0221
ADG, kg	0.42 ± 0.04 ^ab^	0.52 ± 0.03 ^a^	0.34 ± 0.02 ^b^	0.38 ± 0.02 ^b^	0.0011
ADFI, kg	0.81 ± 0.03	0.79 ± 0.03	0.80 ± 0.02	0.79 ± 0.03	0.9155
G:F ratio	0.52 ± 0.04 ^b^	0.66 ± 0.03 ^a^	0.43 ± 0.02 ^b^	0.49 ± 0.04 ^b^	0.0004
FCR	2.01 ± 0.17 ^ab^	1.54 ± 0.09 ^b^	2.39 ± 0.13 ^a^	2.14 ± 0.14 ^a^	0.0023

Values are represented as the mean ± standard error of the mean (SEM). ^1^ CON: control, basal diet; SYN: basal diet + synbiotics (0.2% of bamboo + 0.8% orange peel/kg feed, 10^10^ colony-forming unit (CFU) of Lactobacillus acidophilus + 10^9^ Devosia insulae/animal); DON: basal diet + deoxynivalenol (10 mg/kg feed); and DON+SYN: basal diet + deoxynivalenol (10 mg/kg feed) + synbiotics (0.2% of bamboo + 0.8% orange peel/kg feed, 10^10^ CFU of Lactobacillus acidophilus + 10^9^ Devosia insulae/animal). ^2^ BW, body weight; ADG, average daily gain; ADFI, average daily feed intake; G:F ratio, gain to feed ratio; FCR, feed conversion ratio. ^3^ Measured on day 1 after one week of adaptation. ^4^ Measured on day 28 after one week of adaptation. ^a,b^ Different superscript letters indicate that variables within a row are significantly different (*p* < 0.05).

**Table 2:** Effects of high-dose deoxynivalenol (DON) and synbiotics (SYNs) as DON-detoxifying additives on blood biochemistry of weaned piglets ^1^.

	CON	SYN	DON	DON+SYN	*p* Value
Parameters ^2^	(*n* = 8)	(*n* = 8)	(*n* = 8)	(*n* = 8)	
GLU, mg/dL	101.3 ± 4.2 ^a^	99.9 ± 2.4 ^a^	84.9 ± 3.7 ^b^	102.3 ± 5.9 ^a^	0.0240
CREA, mg/dL	0.8 ± 0.0 ^b^	0.8 ± 0.0 ^b^	0.9 ± 0.0 ^a^	0.9 ± 0.1 ^a^	0.0297
BUN, mg/dL	8.9 ± 1.0	8.4 ± 0.6	7.6 ± 0.8	9.4 ± 0.6	0.3991
BUN/CREA	12.3 ± 1.5	10.5 ± 0.8	9.0 ± 0.8	10.4 ± 0.7	0.1618
PHOS, mg/dL	9.7 ± 0.5	9.8 ± 0.4	9.4 ± 0.3	9.0 ± 0.3	0.4702
CA, mg/dL	8.8 ± 0.2	8.6 ± 0.4	8.4 ± 0.0	8.6 ± 0.1	0.1574
TP, g/dL	4.7 ± 0.1	4.7 ± 0.1	4.9 ± 0.1	4.7 ± 0.1	0.6701
ALB, g/dL	2.4 ± 0.1	2.4 ± 0.1	2.4 ± 0.1	2.4 ± 0.1	0.9957
GLOB, g/dL	2.3 ± 0.1	2.4 ± 0.1	2.5 ± 0.1	2.3 ± 0.1	0.3658
ALB/GLOB	1.0 ± 0.1	1.0 ± 0.1	1.0 ± 0.1	1.0 ± 0.1	0.9227
ALT, U/L	244.1 ± 16.6	222.3 ± 21.1	249.4 ± 31.2	256.0 ± 19.7	0.7467
ALKP, U/L	300.8 ± 26.1	350.3 ± 35.3	381.4 ± 56.5	342.5 ± 25.6	0.5242
GGT, U/L	29.4 ± 2.4	35.3 ± 3.5	34.4 ± 2.4	32.4 ± 4.7	0.6297
TBIL, mg/dL	0.2 ± 0.0	0.2 ± 0.0	0.2 ± 0.0	0.1 ± 0.0	0.7660
CHOL, mg/dL	106.0 ± 7.1 ^a^	108.9 ± 8.2 ^a^	88.3 ± 3.2 ^b^	90.0 ± 4.8 ^b^	0.0481
AMYL, U/L	528.8 ± 87.2	591.8 ± 71.8	573.6 ± 74.3	581.4 ± 71.8	0.9405
LIPA, U/L	37.9 ± 9.4	47.8 ± 13.4	38.0 ± 8.1	44.9 ± 13.3	0.8996

Values are represented as the mean ± standard error of the mean (SEM). ^1^ CON: control, basal diet; SYN: basal diet + synbiotics (0.2% of bamboo + 0.8% orange peel/kg feed, 10^10^ colony-forming unit (CFU) of *Lactobacillus acidophilus* + 10^9^ *Devosia insulae*/animal); DON: basal diet + deoxynivalenol (10 mg/kg feed); and DON+SYN: basal diet + deoxynivalenol (10 mg/kg feed) + synbiotics (0.2% of bamboo + 0.8% orange peel/kg feed, 10^10^ CFU of *Lactobacillus acidophilus* + 10^9^ *Devosia insulae*/animal). ^2^ GLU: glucose; CREA: creatinine; BUN: blood urea nitrogen; PHOS: phosphate; CA: calcium; TP: total protein (TP = ALB + GLOB); ALB, albumin globulin; GLOB, globulin; ALT, alanine aminotransferase; ALKP, alkaline phosphatase; GGT, gamma glutamyl transpeptidase; TBIL, total bilirubin; CHOL, cholesterol; AMYL, amylase; LIPA, lipase. ^a,b^ Different superscript letters indicate that variables within a row are significantly different (*p* < 0.05).

**Table 3:** Stepwise multiple regression analysis for predicting final body weight using the microbiota in the caecum, ileum, and feces of the weaned piglets.

Variables	Regression Coefficient	SE	Partial R^2^	Pr > F
Intercept	26.0921	1.1948		
*Prevotella* 1	−0.8861	0.3103	0.14	0.0380
*Romboutsia*	−0.1341	0.0590	0.27	0.0308
Total R^2^			0.41	

SE: standard error.

## Data Availability

The dataset is available upon request from the authors.
